# Characterization of Glyphosate Resistance and Degradation Profile of *Caballeronia zhejiangensis* CEIB S4-3 and Genes Involved in Its Degradation

**DOI:** 10.3390/microorganisms13030651

**Published:** 2025-03-13

**Authors:** Manuel Isaac Morales-Olivares, María Luisa Castrejón-Godínez, Patricia Mussali-Galante, Efraín Tovar-Sánchez, Hugo Albeiro Saldarriaga-Noreña, Alexis Rodríguez

**Affiliations:** 1 Programa de Doctorado en Ciencias Naturales, Universidad Autónoma del Estado de Morelos, Av. Universidad 1001, Col. Chamilpa, Cuernavaca 62209, Morelos, Mexico; manuel.morales@uaem.edu.mx; 2 Facultad de Ciencias Biológicas, Universidad Autónoma del Estado de Morelos, Av. Universidad 1001, Col. Chamilpa, Cuernavaca 62209, Morelos, Mexico; 3 Centro de Investigación en Biotecnología, Universidad Autónoma del Estado de Morelos, Av. Universidad 1001, Col. Chamilpa, Cuernavaca 62209, Morelos, Mexico; patricia.mussali@uaem.mx; 4 Centro de Investigación en Biodiversidad y Conservación, Universidad Autónoma del Estado de Morelos, Av. Universidad 1001, Col. Chamilpa, Cuernavaca 62209, Morelos, Mexico; efrain_tovar@uaem.mx; 5 Centro de Investigaciones Químicas, Universidad Autónoma del Estado de Morelos, Av. Universidad 1001, Col. Chamilpa, Cuernavaca 62209, Morelos, Mexico; hsaldarriaga@uaem.mx

**Keywords:** bioremediation, genomic analysis, pesticides, AMPA, environmental pollution

## Abstract

Herbicides are the most employed pesticides in agriculture worldwide; among them, glyphosate is the most successful herbicide molecule in history. The extensive use of glyphosate has been related to environmental pollution and toxic effects on non-target organisms. Effective remediation and treatment alternatives must be developed to reduce the environmental presence of glyphosate and its adverse effects. Bioremediation using microorganisms has been proposed as a feasible alternative for treating glyphosate pollution; due to this, identifying and characterizing microorganisms capable of biodegrading glyphosate is a key environmental task for the bioremediation of polluted sites by this herbicide. This study characterized the glyphosate resistance profile and degradation capacity of the bacterial strain *Caballeronia zhejiangensis* CEIB S4-3. According to the results of the bacterial growth inhibition assays on agar plates, *C. zhejiangensis* CEIB S4-3 can resist exposure to high concentrations of glyphosate, up to 1600 mg/L in glyphosate-based herbicide (GBH) formulation, and 12,000 mg/L of the analytical-grade molecule. In the inhibition assay in liquid media, *C. zhejiangensis* CEIB S4-3 resisted glyphosate exposure to all concentrations evaluated (25–400 mg/L). After 48 h exposure, GBH caused important bacterial growth inhibition (>80%) at concentrations between 100 and 400 mg/L, while exposure to analytical-grade glyphosate caused bacterial growth inhibitions below 15% in all tested concentrations. Finally, this bacterial strain was capable of degrading 60% of the glyphosate supplemented to culture media (50 mg/L), when used as the sole carbon source, in twelve hours; moreover, *C. zhejiangensis* CEIB S4-3 can also degrade the primary glyphosate degradation metabolite aminomethylphosphonic acid (AMPA). Genomic analysis revealed the presence of genes associated with the two reported metabolic pathways for glyphosate degradation, the sarcosine and AMPA pathways. This is the first report on the glyphosate degradation capacity and the genes related to its metabolism in a *Caballeronia* genus strain. The results from this investigation demonstrate that *C. zhejiangensis* CEIB S4-3 exhibits significant potential for glyphosate biodegradation, suggesting its applicability in bioremediation strategies targeting this contaminant.

## 1. Introduction

In modern agriculture, various agrochemicals, mainly fertilizers and pesticide compounds, are used extensively in crops. The use of these substances enhances the quality of farm products, increases agricultural production, and controls different pests in crop fields [1,2,3]. Among the pests that threaten crops are the so-called weeds, defined as colonizing plants with an exceptional ability to take advantage of the ecological disturbances caused by humans (e.g., in cropland) [4]. Weeds generate broad impacts on agricultural production; these plant species significantly reduce crop productivity (up to 30% of production, mainly in the least developed countries) because they compete with crops for resources such as soil nutrients (i.e., N, P, and K), space, sunlight, and water, which is why the use of different substances with herbicidal activities is necessary for their control [5,6,7,8]. Herbicides comprise the most widely used group of pesticides in agriculture worldwide. According to Food and Agriculture Organization (FAO) data, in the year 2022, the total pesticide use worldwide was estimated to be 3.7 million tons, of which 1.9 tons (52.6%) corresponds to herbicides [9].

The first attempts to chemically control weeds in crops began in the 1940s with the development of the herbicide 2,4-Dichlorophenoxyacetic acid (2,4-D) [10]. Since then, thousands of formulations that include molecules with herbicidal activity have been marketed worldwide. Among the most widely used herbicides worldwide, glyphosate-based formulations stand out [11,12,13,14]. Glyphosate (N-(phosphonomethyl) glycine) is a synthetic phosphonate molecule with a non-selective herbicide activity widely used to eliminate weeds and facilitate grain harvesting in crop fields worldwide [15,16,17].

The herbicidal activity of glyphosate is based on its ability to inhibit the action of the enzyme 5-enolpyruvyl shikimate-3-phosphate synthase (EPSPS) involved in the shikimate pathway in plants [18,19,20]. EPSPS catalyzes the condensation of shikimate-3-phosphate (S3P) and phosphoenolpyruvate (PEP) molecules to form 5-enolpyruvyl shikimate-3-phosphate (EPSP) [21], a key metabolite for the synthesis of aromatic precursors (chorismate and prephenate) of amino acids such as phenylalanine, tyrosine and tryptophan, and other essential compounds needed for optimal plant development such as folic acid and menaquinone [20]. Due to its chemical similarity to PEP, glyphosate can bind to the interaction site of this molecule in the EPSPS enzyme and inhibit EPSP synthesis [22,23].

Due to its effectiveness in weed control, easy use, and affordable cost, glyphosate use has gradually increased in agriculture. However, the development of herbicide-resistant transgenic crops in 1990 caused a significant rise in glyphosate use worldwide [24]. It is estimated that more than 8.6 million tons of glyphosate (active principle) have been commercialized since its market introduction in 1974; the yearly use of glyphosate is around 785,000 tons, 90% of it employed in agriculture worldwide [25,26], but it is expected that its global use will reach 900,000 tons in 2025 [27]. The extensive use of glyphosate has been related to environmental pollution and toxic effects on non-target organisms such as bacteria, algae, plants, animals and aquatic organisms [28,29,30,31,32,33].

Glyphosate and its primary degradation product, aminomethylphosphonic acid (AMPA), have been detected in soils, as well as in surface and groundwater sources near agricultural regions globally [34,35,36]. In different regions of the European Union, the concentrations of glyphosate and AMPA in agricultural soils may vary from 0.5 to 2 mg/Kg [37]. On the hydroculture side, studies conducted in countries of the European Union and North and South America revealed concentrations in the range of 0.1–328 µg/L in surface water bodies and 0.7 to 2.5 µg/L in groundwater [38].

The presence of glyphosate and its metabolites in the environment is considered a potential ecotoxicological threat [39,40]. Glyphosate exposure may induce acute and chronic toxic effects on non-target organisms distributed in aquatic and terrestrial environments, including microorganisms, plants, animals and humans [41,42,43]. The main toxic effects of glyphosate in humans include (1) direct damage at cellular, tissular and organic levels, (2) oxidative stress through reactive oxygen induction, (3) endocrine disruptor activities, and (4) genetic damage [44].

Due to glyphosate’s environmental and human health threats, developing treatment approaches to eliminate glyphosate and AMPA from the environment and reduce exposure in non-target organisms is needed [45,46]. Several physicochemical alternatives (coagulation, adsorption, reverse osmosis, advanced oxidation processes, among others) have been proposed to deal with glyphosate pollution; however, many of them just remove glyphosate without degradation, could be expensive, difficult to implement and maintain, or could generate secondary pollution [47,48]. Biological methods have demonstrated their efficacy as safe, cost-effective, and dependable strategies for glyphosate removal from soil and water [29,49].

Microorganisms, bacteria and fungi living in polluted environments, such as those impacted with pesticides, could develop genetic and metabolic strategies to degrade these toxic molecules into non-harmful products [50,51]. Bacteria environmentally exposed to glyphosate develop cellular mechanisms to counteract the toxic effects of this pesticide and survive [52]. These mechanisms include a reduction in the number of glyphosate transporters in the bacterial membrane, the expression of efflux proteins, overexpression of the EPSPS enzyme in the presence of glyphosate, mutations in EPSPS to reduce its sensitivity to glyphosate, enzymatic modification of glyphosate, and its biodegradation [16,36,53,54].

During the biodegradation process, the metabolic processes of microorganisms break down glyphosate into smaller molecules. There are two metabolic pathways used by bacteria to degrade glyphosate: in the first, denominated as the sarcosine pathway, the enzyme C-P lyase breaks the carbon–phosphorus bond in glyphosate to produce sarcosine; and in the second, denominated the AMPA pathway, the enzyme glyphosate oxidoreductase breaks glyphosate to produce AMPA [17]. Several bacterial strains have been identified as capable of degrading glyphosate [29,52], including multiple strains from the *Burkholderia* genus, such as *Burkholderia* sp. AQ5-13 [55], *Burkholderia vietnamiensis* AQ5-12 [56], and *Burkholderia cenocepacia* CEIB S5-2 [57]. *Caballeronia* is a bacterial genus closely related to the *Burkholderia* and *Paraburkholderia* genera. In 2016, Dobritsa and Samadpour [58] reclassified and included 12 species of these two bacterial genera in the *Caballeronia* genus, among them *Burkholderia zhejiangensis*.

*Caballeronia zhejiangensis* CEIB S4-3, formerly *Burkholderia zhejiangensis* CEIB S4-3, was isolated from *Tagetes erecta* L. (commonly called cempasúchil in Mexico) crops in Morelos, Mexico. This bacterial strain demonstrates a rapid ability to hydrolyze the organophosphorus pesticide methyl parathion (50 mg/L) and can completely degrade its primary hydrolysis product, *p*-nitrophenol, an extremely toxic compound for microbial communities in soils, within a mere 12 h [59,60]. In this study, the genomic analysis of this strain revealed the presence of genes involved in the two known metabolic pathways for glyphosate degradation in bacteria. Based on this, the objective of the current study was to assess the glyphosate resistance profile of *C. zhejiangensis* CEIB S4-3, as well as its capacity to degrade both glyphosate and AMPA.

## 2. Materials and Methods

### 2.1. Glyphosate

Glyphosate resistance and degradation experiments were performed using a glyphosate-based herbicide formulation (FAENA^®^, 363 g/L, Monsanto Comercial S. de R.L. de C.V., Zapopan, Jalisco, Mexico), high-purity glyphosate (99.5%) analytical reagent, and analytical-grade AMPA (98.0% purity) (Chem Service Inc., West Chester, PA, USA). Stock solutions of the herbicide formulation, glyphosate, and AMPA were prepared at 10,000 mg/L. Finally, appropriate concentrations of these chemicals were adjusted to conduct the experiments.

### 2.2. Bacterial Strain and Culture Media

For pre-inoculum preparation, *C. zhejiangensis* CEIB S4-3 was grown on Petri dishes with Trypticase Soy Agar (TSA), and cultures were incubated for 24 h at 30 °C. Subsequently, individual colonies were transferred into 250 mL Erlenmeyer flasks containing 50 mL of Trypticase Soy Broth (TSB) (Bioxon, Becton Dickinson, Cuautitlán, Estado de Mexico, Mexico) to cultivate the bacterial biomass required for the Minimum Inhibitory Concentration (MIC) assays and bacterial growth inhibition assessments in liquid cultures. For the glyphosate degradation experiments, a Minimal Salt Medium (MSM) was used, which is prepared from two stock solutions. Solution A contains the following per liter: 0.82 g KH_2_PO_4_, 0.19 g K_2_HPO_4_, 0.20 g MgSO_4_·7H2O, 2 g KNO_3_, and 0.99 g (NH_4_)_2_SO_4_. Solution B consists of 2.8 g H_3_BO_3_, 2.55 g MnSO_4_·H_2_O, 0.17 g CuSO_4_·5H_2_O, 2.43 g CoCl_2_·6H_2_O, and 0.25 g ZnSO_4_·7H_2_O. Both solutions were prepared separately, and each was sterilized individually before use. To prepare MSM, 2 mL of Solution B was added to 1 L of Solution A; the final pH of the medium was 7. The MSM was then ready for use in the glyphosate degradation experiments [57].

### 2.3. Glyphosate Bacterial Resistance, Minimum Inhibitory Concentration (MIC) on Agar Plates

To assess glyphosate resistance profile of *C. zhejiangensis* CEIB S4-3 against both commercial and analytical-grade glyphosate, the bacterial strain was cultured in 250 mL Erlenmeyer flasks containing 50 mL of Trypticase Soy Broth (TSB) at 30 °C for 24 h with constant agitation at 150 rpm. Following incubation, bacterial biomass was harvested by centrifugation at 3500 rpm for 10 min. The inoculum was then standardized to an optical density of 0.5 at 600 nm (OD_600nm_), and 1 mL of this inoculum was mixed with 9 mL of warm Tryptic Soy Agar (TSA). The mixture was homogenized and subsequently poured into Petri dishes.

Once solidified, 5 mm diameter filter paper disks were placed on the TSA, each loaded with 20 μL of the corresponding glyphosate solution. After application, the Petri dish was left slightly open for 10 min in the laminar flow hood to allow the filter paper disks loaded with glyphosate to dry before incubation. The inhibitory effects of glyphosate in commercial herbicide formulation and analytical reagent were evaluated at the following concentrations: 25, 50, 100, 200, 400, 800, 1600, 3200, 6400 and 12,000 mg/L, sterile H_2_O (MiliQ) was used as negative inhibition indicator (C−), while the glyphosate-based herbicide (GBH) formulation (363 g/L) was used as a positive indicator (C+) of bacterial inhibition. The Petri dishes were incubated for 24 h at 30 °C, after which the diameter of developed inhibition halos in the Petri dishes were measured. Three biological replicates were conducted for each experiment.

### 2.4. Bacterial Growth Inhibition Assays in Trypticase Soy Broth (TSB) Medium

To evaluate the effect of glyphosate exposure on bacterial growth in liquid cultures, *C. zhejiangensis* CEIB S4-3 was grown in 50 mL of TSB at 30 °C for 24 h and 150 rpm for pre-inoculum preparation. Subsequently, the bacterial biomass was collected by centrifugation at 3500 rpm for 10 min and the experimental inoculum was adjusted to 0.05 OD_600nm_. For inhibition assays, 250 mL Erlenmeyer flasks were inoculated with 50 mL of TSB supplemented with different concentrations of glyphosate in the GBH commercial formulation and the analytical-grade glyphosate at the following concentrations of 25, 50, 100, 200, and 400 mg/L. The OD_600nm_ of the bacterial cultures without glyphosate was considered reference bacterial growth, while TSB was used as a blank. The cultures were incubated for 24 h at 30 °C and 150 rpm, and bacterial growth was assessed through the OD_600nm_ every two hours until 24 h. Three biological replicates were conducted for each experiment.

### 2.5. Bacterial Growth Inhibition Assays in Minimal Salts Medium (MSM)

To evaluate the effect of glyphosate exposure on bacterial growth in liquid cultures using glyphosate as sole carbon source, after pre-inoculum preparation, *C. zhejiangensis* CEIB S4-3 (0.5 OD_600nm_) was grown in 250 mL Erlenmeyer flasks with 50 mL of MSM at pH 7 supplemented with 25, 50, 100, 200 and 400 mg/L of GBH formulation and analytical-grade glyphosate. The cultures were incubated for 24 h at 30 °C and 150 rpm, and the optical density (OD_600nm_) of the cultures was assessed every two hours until 24 h. Three biological replicates were conducted for each experiment.

### 2.6. Glyphosate Degradation Kinetics

To evaluate glyphosate degradation capability of *C. zhejiangensis* CEIB S4-3, degradation kinetics were conducted. The bacterial strain was inoculated (0.5 OD_600nm_) in 250 mL Erlenmeyer flasks with 50 mL of MSM supplemented with a concentration of 50 mg/L of glyphosate (analytic reagent). Cultures were incubated at 30 °C at 150 rpm. Every two hours, samples of 1.5 mL were collected until 8 h; the samples were centrifuged at 4000 rpm for 10 min and filtered through a 0.2 μm poly(vinylidene fluoride) (PVDF) filter (CHROMAFIL^®^Xtra, MachenreyNagel, Düren, Germany). Glyphosate degradation was analyzed using Ultra-Performance Liquid Chromatography-Tandem Mass Spectrometry (UPLC-MS/MS) as described by Roy et al. (2021) [61]. Glyphosate and AMPA residues in the culture supernatants were quantified using a Liquid Chromatograph (Agilent 1290 Infinity II LC system, Santa Clara, CA, USA) coupled with a mass spectrometer (Agilent 6545 Q-TOF, Santa Clara, CA, USA). The separation of metabolites was performed via UPLC on a C_18_ ACE Excel 2 C18-PFP column (2 μm, 2.1 mm × 150 mm; HiChrom, Reading, UK), with an isocratic flow rate of 0.35 mL/min, using water (0.1% formic acid) and methanol (0.1% formic acid) as mobile phases. The eluted glyphosate and AMPA metabolites were then identified through mass spectrometry.

### 2.7. Genomic Analyses

To investigate potential genes involved in the biodegradation of glyphosate within the *C. zhejiangensis* CEIB S4-3 genome, bioinformatic tools were employed to analyze the draft genome of this bacterial strain, which is publicly accessible on NCBI (https://www.ncbi.nlm.nih.gov/ (accessed on 22 January 2025) under BioProject accession number PRJNA264584 [62]. The search for genes encoding enzymes associated with the glyphosate degradation pathways (sarcosine and AMPA) was conducted by aligning the amino acid sequences of orthologous enzymes from related bacterial genera, including *Burkholderia*, *Caballeronia*, and *Pseudomonas*. Gene identification was performed by translating the protein sequences into their corresponding nucleotide sequences, followed by sequence alignments using the TBlastn tool in the Basic Local Alignment Search Tool (BLAST, https://blast.ncbi.nlm.nih.gov/Blast.cgi (accessed on 22 January 2025).

### 2.8. Degradation Rate of Glyphosate (mg/L·h)

The wide variety of pesticide molecules, bacterial species, and initial glyphosate concentrations across the identified studies complicates the comparison and identification of the most effective systems for glyphosate degradation. To address this, it was suggested to calculate the glyphosate degradation rate for different bacterial species in order to pinpoint those with the highest efficiency in degrading glyphosate using the following formula:

GDR = I_c_ − F_c_/Dt (1)

where:

GDR: Glyphosate degradation rate (mg/L·h)
Ic: Initial concentration (mg/L)
Fc: Final concentration (mg/L)
Dt: Degradation time (h)

### 2.9. Statistical Analyses

The data obtained from the experimental results were processed using a Shapiro-Wilk test to assess the normality of the data, and a Bartlett test was performed to determine the homogeneity of variance of the data. Subsequently, once the normality of the data and the homogeneity of variance were determined, a two-way analysis of variance (ANOVA) was performed to assess the effect of glyphosate at different concentrations on the growth of the bacterial strain. At the same time, a Tukey test was performed to determine significant differences in the growth of the bacterial strain due to exposure to glyphosate over time. Significant differences in the concentration of glyphosate and AMPA across time in the glyphosate degradation kinetics was determined through one-way ANOVA and Tukey test.

## 3. Results

### 3.1. Glyphosate Bacterial Resistance, Minimum Inhibitory Concentration (MIC) on Agar Plates

**Figure 1:**
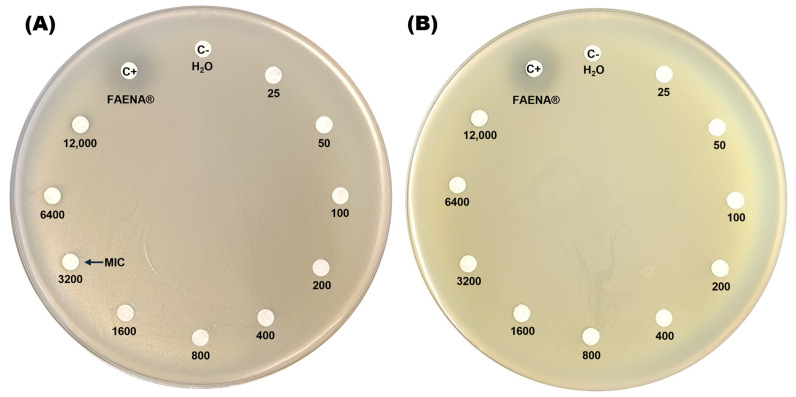
Determination of the minimal inhibitory concentration (MIC) of glyphosate on agar plates. Panel (**A**) shows *C. zhejiangensis* CEIB S4-3 exposed to the commercial glyphosate-based herbicide (GBH) formulation (FAENA^®^), while panel (**B**) illustrates the exposure to analytical-grade glyphosate. Sterile water (MilliQ) served as the negative control (C−), while a concentrated GBH solution (363 g/L) was used as the positive control (C+) in both experiments.

The resistance profile of the *C. zhejiangensis* CEIB S4-3 strain to glyphosate was assessed by exposing the bacteria to increasing concentrations of both a commercial GBH formulation (FAENA^®^) and analytical-grade glyphosate (98% purity) on agar plates. In the minimal inhibitory concentration test on agar plates, the GBH formulation displays inhibition halos at concentrations in the range of 3200–12,000 mg/L (Figure 1A), while in the plates with the presence of analytical-grade glyphosate, no inhibition halos were observed in the experiments (Figure 1B). The findings indicate that *C. zhejiangensis* CEIB S4-3 can tolerate exposure to up to 1600 mg/L of glyphosate in its commercial formulation (GBH). In contrast, the bacterial strain demonstrated resistance to all concentrations of analytical-grade glyphosate tested.

### 3.2. Bacterial Growth Inhibition Assays in Trypticase Soy Broth (TSB) Medium

**Figure 2:**
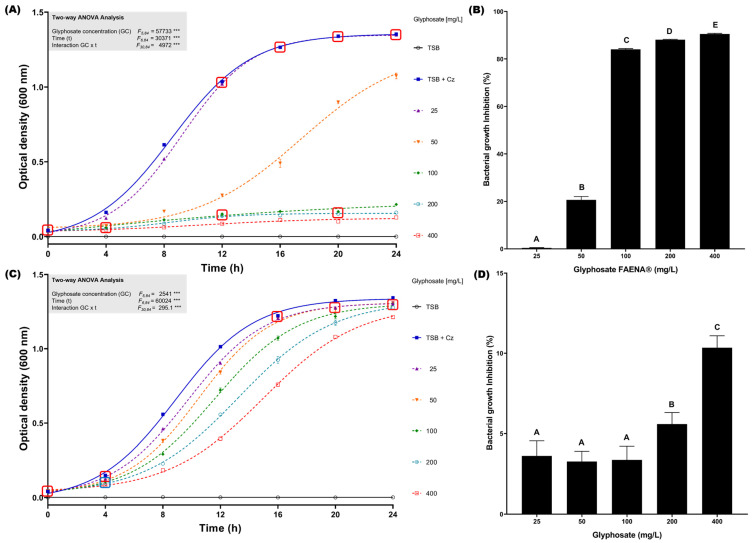
Glyphosate resistance kinetics in TSB medium. (**A**) *C. zhejiangensis* CEIB S4-3 (Cz) exposed to GBH formulation (FAENA^®^); the experimental points inside the squares do not show significant statistical differences. (**B**) Bacterial growth inhibition percentages in cultures exposed to GBH formulation; the bars with the same letters do not show significant statistical differences. (**C**) *C. zhejiangensis* CEIB S4-3 exposed to analytical-grade glyphosate; the experimental points inside the squares do not show significant statistical differences. (**D**) Bacterial growth inhibition percentages in cultures exposed to analytical-grade glyphosate; the bars with the same letters do not show significant statistical differences. Concentrations of 50–400 mg/L were used in the experiments. Three biological replicates were conducted in all experiments; point and bars represent average values, and error bars show the standard deviation. Two-way ANOVA test: *** = *p* ≤ 0.001.

To determine the glyphosate resistance profile of *C. zhejiangensis* CEIB S4-3 in liquid cultures, growth kinetics in the presence of concentrations from 25 to 400 mg/L of glyphosate in the commercial GBH formulation and analytical-grade reagent were conducted at 24 h (30 °C and 150 rpm). In the assay with the presence of glyphosate in commercial GBH formulation, *C. zhejiangensis* CEIB S4-3 was capable of resisting all concentrations evaluated (Figure 2A); however, exposure to concentrations of 100, 200, and 400 mg/L caused important growth inhibition of 84.1 ± 0.3, 88.1 ± 0.2 and 90.5 ± 0.3%, respectively (Figure 2B), whereas when the bacterial strain was exposed to a concentration of 50 mg/L the observed growth inhibition was just 20.6 ± 1.6%. Finally, exposure to 25 mg/L did not cause a significant inhibition in bacterial growth (0.4 ± 0.1%); the bacterial growth profile did not show significant differences concerning the kinetics in the absence of glyphosate after 12 h incubation (Figure 2A). In the experiments conducted with analytical-grade glyphosate, *C. zhejiangensis* CEIB S4-3 showed lower growth inhibition with respect to experiments with GBH formulation (Figure 2C). The highest growth inhibition (10.3 ± 0.8%) was observed in the exposure to 400 mg/L of glyphosate (analytical grade), while growth inhibitions were below 5% at concentrations from 25–100 mg/L (Figure 2D). According to these findings, the bacterial strain can resist glyphosate exposure at concentrations of 25 and 50 mg/L in the GBH formulation and 25–400 mg/L when the bacterial strain is exposed to analytical-grade glyphosate.

### 3.3. Bacterial Growth Inhibition Assays in Minimal Salts Medium (MSM)

**Figure 3:**
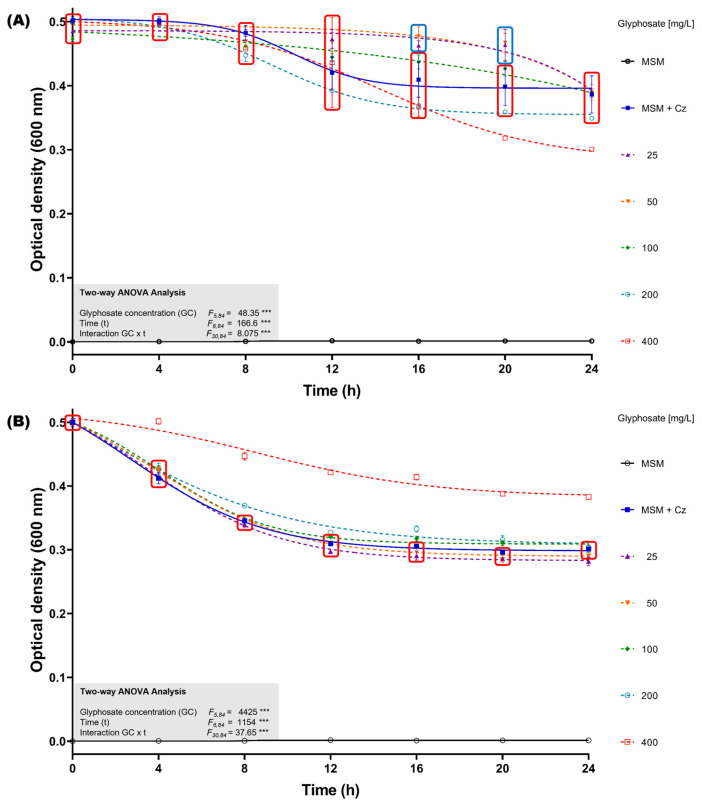
Glyphosate resistance kinetics in MSM medium. (**A**) *C. zhejiangensis* CEIB S4-3 (Cz) exposed to GBH formulation (FAENA^®^). (**B**) *C. zhejiangensis* CEIB S4-3 exposed to analytical-grade glyphosate. The experimental points inside the squares do not show significant statistical differences. Concentrations of 50–400 mg/L were used in the experiments. Three biological replicates were conducted in all experiments; point and bars represent average values, and error bars show the standard deviation. Two-way ANOVA test: *** = *p* ≤ 0.001.

To assess the impact of glyphosate exposure without the presence of supplementary carbon sources in the culture medium, bacterial growth inhibition assays were performed in minimal salts medium (MSM) supplemented with glyphosate concentrations ranging from 25 to 400 mg/L, using both the GBH formulation and analytical-grade glyphosate. The initial optical density at 600 nm (OD_600nm_) of the *C. zhejiangensis* CEIB S4-3 cultures was standardized to 0.5. When the bacterial strain was exposed to the GBH formulation, it was observed that in concentrations from 25–100 mg/L, the culture showed no growth inhibition up to 16 h (Figure 3A); after that time, a reduction in the OD_600nm_ of the cultures was observed, eventually reaching similar OD_600nm_ values to those observed in the experiment in the absence of glyphosate at the end of the kinetics. Otherwise, exposure to concentrations of 200 and 400 mg/L caused a reduction in the OD_600nm_ of the cultures when compared to the experiment in the absence of glyphosate; the highest inhibition (22.2 ± 0.1%) was observed at 400 mg/L (Figure 3B).

The exposure of *C. zhejiangensis* CEIB S4-3 to analytical-grade glyphosate showed different results compared to the experiments using the GBH formulation. Exposure to concentrations from 25 to 200 mg/L did not cause important changes in the OD_600nm_ of the cultures with respect to the experiments in the absence of glyphosate; however, an increase in OD_600nm_ of the culture was observed at a concentration of 400 mg/L (Figure 3B). These findings suggest that *C. zhejiangensis* CEIB S4-3 can use glyphosate as a carbon source for bacterial maintenance.

### 3.4. Glyphosate Degradation Kinetics

**Figure 4:**
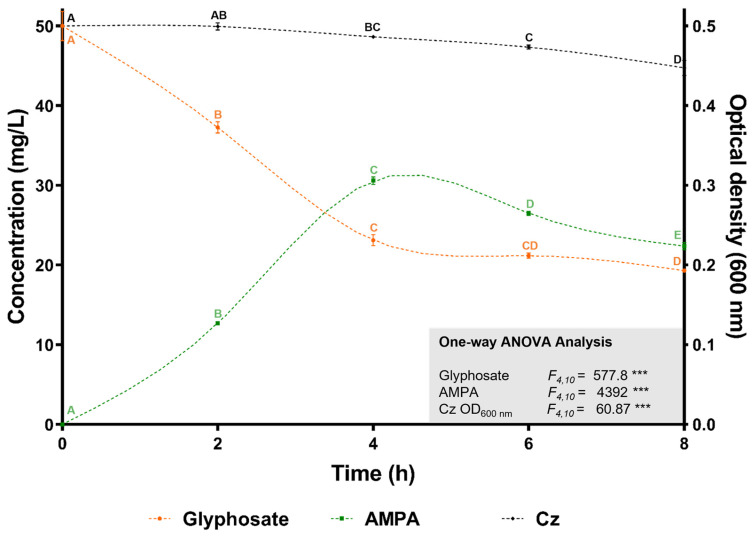
Glyphosate degradation kinetics. *C. zhejiangensis* CEIB S4-3 (Cz) was inoculated at an initial OD_600nm_ of 0.5 in Mineral Salts Medium (MSM) containing 50 mg/L of glyphosate. Experimental points with the same letters do not show significant statistical differences. One-way ANOVA: *** = *p* ≤ 0.001.

The ability of *C. zhejiangensis* CEIB S4-3 to degrade glyphosate was assessed in shaken flasks (30 °C, 150 rpm) containing 50 mg/L of analytical-grade glyphosate in Mineral Salts Medium (MSM), with bacterial inoculation at an initial OD_600nm_ of 0.5. Glyphosate degradation was monitored every two hours over an eight-hour period. As shown in Figure 4, the initial glyphosate concentration decreased progressively over the course of the experiment. After eight hours, the glyphosate concentration was significantly reduced to 19.3 ± 0.2 mg/L, reflecting a degradation rate of 61.4 ± 0.4%. The release of aminomethylphosphonic acid (AMPA) was detected after two hours of incubation, reaching its peak concentration of 15.3 ± 0.23 mg/L at four hours, before declining to 11.8 ± 0.24 mg/L by the end of the experiment. These results demonstrate that *C. zhejiangensis* CEIB S4-3 not only tolerates high concentrations of glyphosate but also degrades up to 61% of the herbicide in the absence of additional carbon sources. Furthermore, the detection of AMPA suggests the involvement of the glyphosate oxidase enzyme (GOX; E.C. 1.5.3.23) and supports the utilization of the AMPA metabolic pathway for glyphosate degradation by *C. zhejiangensis* CEIB S4-3 (Figure 4).

### 3.5. Genomic Analyses

The genomic analysis of the *C. zhejiangensis* CEIB S4-3 strain enabled the identification of genes encoding enzymes involved in two well-characterized glyphosate degradation pathways, namely the sarcosine and AMPA pathways, as reported in various bacterial species. According to the genomic analysis results, this is the first report on the glyphosate degradation capacity and the genes related to its metabolism in a *Caballeronia* genus strain.

#### 3.5.1. Identification of Genes Implicated in Glyphosate Degradation (Sarcosine Pathway)

Glyphosate degradation through the sarcosine pathway involves two primary enzymatic reactions: (1) the hydrolytic cleavage of the glyphosate molecule to release a phosphate group and produce sarcosine, catalyzed by carbon-phosphorus lyase (C-P lyase) (E.C. 4.7.1.1), and (2) the oxidation of sarcosine to formaldehyde and glycine, catalyzed by the heterotetrameric enzyme sarcosine oxidase (SOX) (E.C. 1.5.3.1; E.C. 1.5.3.24). The resulting products, formaldehyde and glycine, are subsequently utilized in microbial metabolism, with formaldehyde being processed through the tetrahydrofolate pathway and glycine contributing to protein synthesis [29,52].

**Figure 5:**
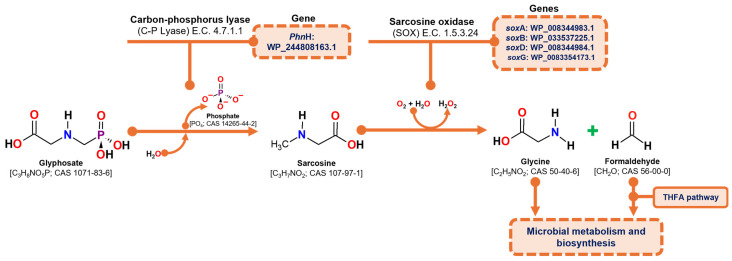
Overview of the sarcosine degradation pathway and the corresponding genes identified in the draft genome of *C. zhejiangensis* CEIB S4-3. THFA represents tetrahydrofolate.

According to the genome analysis of the *C. zhejiangensis* CEIB S4-3 strain, the genes corresponding to the C-P lyase (*Phn*H: WP_244808163.1) and the genes corresponding to the four subunits of sarcosine oxidase were identified: alpha subunit (*sox*A WP_008344983.1), beta (*sox*B WP_033537225.1), delta (*sox*D WP_008344984.1) and gamma (*sox*G WP_008354173.1), so the strain has the genes of the metabolic machinery for the degradation of glyphosate through the sarcosine pathway (Figure 5).

#### 3.5.2. Identification of Genes Involved in Glyphosate Degradation (AMPA Pathway)

The degradation of glyphosate via the AMPA pathway begins with the oxidation of the glyphosate molecule by glyphosate oxidase (GOX) (E.C. 1.5.3.23), resulting in the release of glyoxylate and AMPA. Glyoxylate is then metabolized through the tricarboxylic acid cycle (TCA), where its complete conversion to CO_2_ generates reducing power and ATP. In contrast, AMPA may either be excreted from the cell into the environment or undergo further degradation through two distinct pathways. In the first pathway, C-P lyase (E.C. 4.7.1.1) hydrolyzes the phosphate group from AMPA, releasing methylamine. Methylamine is then oxidized to formaldehyde by methylamine dehydrogenase (E.C. 1.4.9.1), and the resulting formaldehyde enters microbial metabolism via the tetrahydrofolate pathway. In the second pathway, AMPA undergoes a transamination reaction mediated by an aminotransferase enzyme (E.C. 2.6.1.2), transferring its amino group to pyruvic acid and producing phosphoformaldehyde. The phosphate group of phosphoformaldehyde is then hydrolyzed by phosphonatase (E.C. 3.11.1.1), releasing formaldehyde, which is subsequently incorporated into the tetrahydrofolate pathway for further metabolism [29,52].

**Figure 6:**
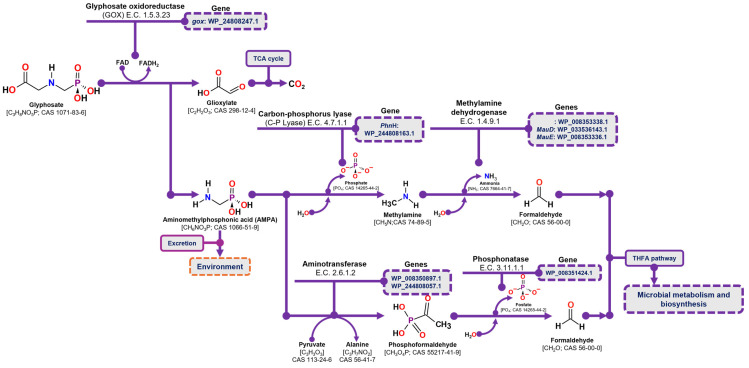
AMPA degradation pathway and the corresponding genes identified in the draft genome of *C. zhejiangensis* CEIB S4-3. TCA refers to the tricarboxylic acid cycle, and THFA denotes tetrahydrofolate.

In the case of glyphosate degradation pathways through AMPA, the *C. zhejiangensis* CEIB S4-3 strain presents in its genome the gene corresponding to the glyphosate oxidase enzyme (*gox*: WP_244808247.1), the C-P lyase (*Phn*H: WP_244808163.1) and three genes related to methylamine metabolism: the amino dehydrogenase (WP_008353338.1), and two genes encoding dehydrogenases involved in methylamine utilization were identified: MauD (WP_033536143.1) and MauE (WP_008353336.1). Likewise, *C. zhejiangensis* CEIB S4-3 presents genes that encode enzymes related to the alternative pathway for AMPA degradation, mediated by a transamination process, including two genes that encode aminotransferases, such as the 2-aminoethylphosphonate aminotransferase gene (WP_008350897.1) and the class II aminotransferase gene, dependent on pyridoxal phosphate (WP_244808057.1), and a gene that encodes a phosphonatase (WP_008351424.1) (Figure 6). In accordance with the above, the *C. zhejiangensis* CEIB S4-3 strain presents in its genome the key enzymes for AMPA metabolism and its conversion to formaldehyde, for its use through the tetrahydrofolate cycle.

## 4. Discussion

The shikimate pathway plays a critical role in the development of plants, as well as in microorganisms, including algae, bacteria and fungi. Consequently, the inhibition of the enzyme EPSP by glyphosate also impacts microbial communities in agricultural soils [63]. Glyphosate exposure has been shown to negatively affect bacterial growth. In a pioneering study, Busse et al. (2001) [64] reported the detrimental effects of glyphosate on the growth of cultivable bacteria and fungi in soil samples collected from *Pinus ponderosa* Douglas & Lowson plantations in California, USA. At a concentration of 8.45 mg/mL (50 mM), glyphosate significantly reduced microbial growth. Additionally, glyphosate exposure has been associated with a reduction in nitrogen fixation rates, likely due to its adverse impact on the number, size, and weight of nitrogen-fixing bacterial nodules [65]. Furthermore, glyphosate-based herbicides often contain surfactants such as polyethoxylated amines (POEAs), which amplify the negative effects of glyphosate on microorganisms [66,67].

As demonstrated in Figure 1, the minimal inhibitory concentration assay for glyphosate on agar plates revealed that exposure to the GBH formulation (FAENA^®^) resulted in bacterial growth inhibition at concentrations exceeding 1600 mg/L. Additionally, in bacterial growth inhibition assays conducted in TSB medium, exposure to the GBH formulation caused a significant reduction in bacterial growth at concentrations of 100, 200, and 400 mg/L (Figure 2A). However, at concentrations of 25 and 50 mg/L, bacterial growth was less affected, indicating a notable resistance profile at these glyphosate concentrations. It is possible that at lower concentrations, the toxic effects of the co-formulants in the commercial glyphosate formulation are diminished. Furthermore, certain bacteria may utilize glyphosate as a source of carbon, nitrogen, and phosphorus, which could contribute to the persistence of microbial growth [45,68,69].

The study of bacteria resistant or tolerant to glyphosate has become a significant area of interest, particularly for the development of bioremediation approaches targeting glyphosate contamination [70]. Bacterial strains isolated from various environments exposed to glyphosate have demonstrated the ability to both withstand and degrade various formulations of this herbicide. These bacteria primarily utilize glyphosate as a source of phosphorus, although certain strains are capable of using it as a nitrogen and carbon source as well [47]. For instance, the Gram-negative bacteria *Acetobacter* sp. G ADA3 and *Pseudomonas fluorescens* G AKL5, which were isolated from rice fields in Nigeria, exhibited tolerance to glyphosate concentrations of up to 250 mg/mL in liquid media. However, at a concentration of 25 mg/mL, the growth of these strains was similar to the control culture at 7.2 mg/mL, which corresponds to the typical field application rate of glyphosate [71]. In a study by Massot et al. (2021), the resistance of bacteria sourced from the rhizosphere of *Lotus* species and pastureland soils in agricultural plots with prolonged glyphosate use was assessed. The observed minimum inhibitory concentrations (MIC) ranged from 500 to 10,000 mg/Kg, with bacterial isolates from the genera *Rhizobium* and *Ochrobactrum* showing tolerance to the highest glyphosate concentration tested (10,000 mg/Kg) [72].

Several studies have emphasized the presence of microorganisms capable of degrading glyphosate in the rhizosphere of plants. Kryuchkova et al. (2014) [73] isolated a bacterial strain, *Enterobacter cloacae* K7, capable of growth in a culture medium containing 1691 mg/L (10 mM) of glyphosate and also capable of degrading 40% of the glyphosate supplemented (845.5 mg/L; 5 mM) in 120 h; glyphosate-degrading activity was attributed to C-P lyase activity, due to the identification of sarcosine and glycine, key intermediate metabolites in the sarcosine pathway. Similarly, Massoti et al. (2021) [74] studied the rhizosphere of *Glycine max* from agricultural fields treated with glyphosate-based herbicides (GBH) for 15–20 years. They isolated the strain *Achromobacter insolitus* SOR2 of the order Burkholderiales, which could grow in MSM supplemented with 253.6 mg/L of glyphosate and degrade the herbicide with 40% efficiency in 96 h. In a more recent study, Nikmah and Lisdiana (2024) [75] isolated 14 bacterial strains from the rhizosphere of *Capsicum frutescens* L.; strains Cf2, Cf3, Cf6, Cf11, Cf12, Cf13, and Cf14 were capable of tolerating and growing in MSM supplemented with 50 mg/L of glyphosate. Subsequently, Lisdiana et al. (2025) [76] sequenced the 16S rRNA gene of strain Cf2, which showed a 98.88% similarity to *Bacillus subtilis*. Both studies suggest that the *Bacillus subtilis* Cf2 strain has potential for glyphosate degradation and could be used in agricultural soils contaminated with this herbicide. Microorganisms frequently exposed to glyphosate may develop adaptive mechanisms, such as upregulating the production of the EPSP synthase enzyme or employing efflux systems to limit intracellular glyphosate accumulation [52,77,78,79].

Despite the known negative impacts of glyphosate on microbial communities, various bacterial strains have been identified as capable of degrading glyphosate under different experimental conditions (Table 1). Among the most commonly reported bacterial genera involved in glyphosate degradation are *Achromobacter*, *Bacillus*, *Ochrobactrum*, and *Pseudomonas*. Additionally, recent studies have highlighted the potential of certain bacterial strains within the *Burkholderia* genus, such as *B. vietnamiensis* AQ5-12 and *Burkholderia* sp. AQ5-13, both isolated from rice fields in Malaysia with extensive herbicide use, to biodegrade glyphosate [55,56]. However, the specific metabolic pathways responsible for this degradation remain undefined. Furthermore, *B. cenocepacia* CEIB S5-2, isolated from agricultural soils in Morelos, Mexico, has been shown to resist and degrade glyphosate through the AMPA pathway [57].

In resistance assays conducted using minimal salt medium (MSM), the optical density (OD_600nm_) of *C. zhejiangensis* CEIB S4-3 increased when exposed to 200 and 400 mg/L of analytical-grade glyphosate (Figure 3B). These findings suggest that analytical-grade glyphosate did not exhibit toxicity toward this strain and indicate the potential for *C. zhejiangensis* CEIB S4-3 to utilize glyphosate as an alternative source of carbon, nitrogen, or phosphorus, thereby sustaining its metabolic activity. Biodegradation assays revealed that *C. zhejiangensis* CEIB S4-3 could degrade 61% of 50 mg/L glyphosate within eight hours, with the release of AMPA detected during the degradation process. The strain was also capable of degrading AMPA itself. While many studies have reported bacterial strains capable of hydrolyzing glyphosate, fewer have demonstrated the ability to incorporate AMPA into cellular metabolism [80]. As a result, AMPA often accumulates in soil, posing a potential environmental concern. The current study, however, observed the degradation of both glyphosate and AMPA, reinforcing the suitability of bacterial strains isolated from pesticide-impacted environments for bioremediation applications.

Biodegradation of glyphosate by either native or non-native microorganisms represents a promising approach for its elimination from contaminated soils and aquatic environments. Numerous studies indicate that certain bacterial species are capable of utilizing glyphosate as a phosphorus source, suggesting the presence of enzymes that can break the C-P bond within the compound. Additionally, some bacteria, such as *Arthrobacter sp. GLP 1/Nit* [81], utilize glyphosate as a nitrogen source. In contrast, other bacteria, including *Streptomyces* sp. StC and *Achromobacter* sp. LW9 [49], degrade glyphosate to derive carbon. There are two primary enzymatic pathways for glyphosate degradation identified in bacterial species.

The initial metabolic route identified for glyphosate degradation is termed the sarcosine pathway. In this process, the direct cleavage of the C-P bond of glyphosate results in the formation of sarcosine and inorganic phosphorus (Pi). This reaction is facilitated by a multi-enzyme complex called C-P lyase. Well-characterized in *Escherichia coli*, the C-P lyase complex consists of 14 genes located within the *phn* operon. Bacterial strains harboring this complex exhibit high efficiency in herbicide degradation under controlled laboratory conditions, particularly when grown in a mineral medium with glyphosate as the exclusive phosphorus source [81]. However, in natural settings, the degradation efficiency is often lower, as the expression of the C-P lyase complex is typically induced only under conditions of intracellular Pi scarcity and phosphorus specificity deficiency, which are not commonly encountered in the environment [49]. The second pathway, known as the AMPA pathway, involves the cleavage of the C-N bond, a process mediated by the enzyme glyphosate oxidase. This leads to the production of glyoxylate and AMPA. In many glyphosate-degrading bacterial species, glyoxylate is utilized as an energy source through its incorporation into the glyoxylate cycle [82,83], while AMPA is usually exported into the extracellular space.

Table 1 presents 46 bacterial strains identified as capable of glyphosate degradation, including *C. zhejiangensis* CEIB S4-3, which was evaluated in this study. For 22 of these strains, the metabolic pathways utilized for glyphosate degradation have been documented: two strains utilize the sarcosine pathway, 14 bacterial strains employ the AMPA pathway, and six strains are capable of using both pathways. The studies summarized in Table 1 report a wide range of glyphosate concentrations (from 20 to 5072 mg/L) and degradation rates (ranging from 20 to 100%), making it difficult to directly compare the efficiency of glyphosate degradation among these bacterial strains. To facilitate comparison, this study calculated the glyphosate degradation rate (GDR, mg/L·h) for each strain. The strains were then ranked based on their degradation rates, from highest to lowest, and divided into quartiles, each containing 12 bacterial strains. The first quartile includes strains with the highest glyphosate degradation rates (2.6–14.4 mg/L·h), the second quartile consists of strains with upper-middle degradation rates (1–2.1 mg/L·h), the third quartile includes strains with lower-middle degradation rates, and the fourth quartile contains strains with the lowest degradation rates, ranging from 0.01 to 0.7 mg/L·h.

Among the strains in the first quartile, which exhibit the highest degradation rates (9.4–14.4 mg/L·h), are *Comamonas odontotermitis* P2, *Rhizobium* sp. SRG, *Sinorhizobium Saheli* SRI, *Ensifer* sp. SR, *Pseudomonas putida* DA, and *Pseudomonas putida* X. *C. zhejiangensis* CEIB S4-3 demonstrated a degradation of 61% of glyphosate (50 mg/L) within eight hours and was also able to eliminate AMPA. With a glyphosate degradation rate of 2.6 mg/L·h, *C. zhejiangensis* CEIB S4-3 is classified in the first quartile, indicating that it is one of the more effective glyphosate degraders, although its degradation rate is lower than that of the top-performing strains. Notably, the strains *Rhizobium* sp. SRG, *S. Saheli* SRI, and *Ensifer* sp. SR utilized glucose (3.6 g/L) as a co-substrate in their degradation processes, while *B. vietnamiensis* AQ5-12 incorporated fructose and ammonium sulfate to enhance glyphosate degradation. These findings suggest that the inclusion of supplementary carbon sources could enhance glyphosate degradation efficiency, a strategy that could be explored to optimize degradation processes. When comparing the glyphosate degradation efficiency of *C. zhejiangensis* CEIB S4-3 with other *Burkholderia* strains, it exhibited a similar degradation rate to *B. vietnamiensis* AQ5-12 but showed a higher rate than *Burkholderia* sp. AQ5-13 (0.6 mg/h) and a lower rate than *B. cenocepacia* CEIB S5-2 (6.3 mg/L·h).

**Table 1:** Recent investigations on glyphosate degradation by bacterial species.

Bacterial Strain	Concentration (mg/L)	Assay Duration (h)	Degradation (%)	Degradation Rate (mg/L·h)	Degradation Pathway	Reference
*Comamonas odontotermitis* P2	1500	104	100	14.4	AMPA & Sarcosine	[84]
*Rhizobium* sp. SRG *	5072	168	44	13.3	-	[85]
*Pseudomonas putida* HE	1800	96	70	13.1	-	[86]
*Sinorhizobium saheli* SRI *	5072	168	40.8	12.3	-	[85]
*Ensifer* sp. SR *	5072	168	38.7	11.7	-	[85]
*Pseudomonas putida* DA	1800	96	50	9.4	-	[86]
*Pseudomonas putida* X	1800	96	50	9.4	-	[86]
*Burkholderia cenocepacia* CEIB S5-2	50	8	100	6.3	AMPA	[57]
*Ochrobactrum intermedium* Sq20	500	104	100	4.8	AMPA & Sarcosine	[79]
*Enterobacter cloacae* K7	845.5	120	40	2.8	Sarcosine	[73]
*Burkholderia vietnamiensis* AQ5-12 ^‡^	100	36	92.3	2.6	-	[56]
*Caballeronia zhejiangensis* CEIB S4-3	50	12	61.1	2.6	AMPA	This work
*Chryseobacterium* sp. Y16C	200	96	100	2.1	AMPA	[87]
*Ochrobactrum anthropi* GPK 3 ^§^	500	150	56	1.9	-	[88]
*Pseudomonas* sp. GC04	500	168	62.7	1.9	AMPA	[89]
*Pseudomonas* sp. GA07	500	168	54.6	1.6	AMPA	[89]
*Ochrobactrum haematophilum* SR	254	96	56	1.5	AMPA	[74]
*Agrobacterium tumefaciens* CHLDO	254	96	47	1.2	AMPA	[74]
*Pseudomonas alcaligenes* Z1–1	200	168	100	1.2	AMPA	[90]
*Achromobacter insolitus* SOR2	253.6	96	40	1.1	AMPA	[74]
*Achromobacter xylosoxidans* SOS3	253.6	96	41	1.1	AMPA	[74]
*Pseudomonas* sp. GA09	500	168	35.5	1.1	AMPA & Sarcosine	[89]
*Achromobacter denitrificans* SOS5	254	96	37	1.0	AMPA	[74]
*Achromobacter* sp. MPK 7A ^§^	500	200	40	1.0	Sarcosine	[88]
*Rhizobium leguminosarum* GP2	250	336	87.6	0.7	AMPA & Sarcosine	[91]
*Stenotrophomonas acidaminiphila* Y4B	50	72	98	0.7	AMPA	[92]
*Bacillus subtilis* GP1	250	336	89.8	0.7	AMPA & Sarcosine	[91]
*Lysinibacillus sphaericus* ^†^	679	720	79	0.7	AMPA	[93]
*Burkholderia* sp. AQ5-13	50	60	74	0.6	-	[55]
*Streptomyces* sp. GP3	250	336	86.2	0.6	AMPA & Sarcosine	[91]
*Bacillus cereus* 6P	169	240	37.7	0.3	-	[94]
*Ochrobactrum* sp. BTU1	100	96	20	0.2	AMPA	[95]
*Enterobacter ludwigii* WAG11	100	672	99.6	0.15	-	[70]
*Pseudomonas aeruginosa* WAG9	100	672	99.4	0.15	-	[70]
*Enterobacter cloacae* WAG5	100	672	95.9	0.14	-	[70]
*Klebsiella variicola* WAG4	100	672	96	0.14	-	[70]
*Serratia liquefaciens* WAG2	100	672	94.1	0.14	-	[70]
*Ochrobactrum* sp. B18	50	360	70	0.1	-	[96]
*Ochrobactrum* sp. DGG-1-3	50	360	60	0.08	-	[96]
*Ochrobactrum* sp. Ge-14	50	360	60	0.08	-	[96]
*Pseudomonas citronellolis* ADA-23B	50	360	60	0.08	-	[96]
*Bacillus megaterium*	25	1440	71	0.01	-	[97]
*Azotobacter* sp.	20	1440	80	0.01	-	[98]
*Bacillus megaterium*	20	1440	87.3	0.01	-	[98]
*Bacillus subtilis*	20	1440	75.1	0.01	-	[98]
*Rhizobium* sp.	20	1440	80	0.01	-	[98]

* Glucose added as a co-substrate at a concentration of 3.6 g/L. ^‡^ Fructose included as a co-substrate at 6 g/L, with ammonium sulfate (0.5 g/L) as the nitrogen source. ^§^ Glutamate used as a co-substrate at 10 g/L. ^†^ Degradation of glyphosate in soil.

## 5. Conclusions

The results of this study demonstrate that *C. zhejiangensis* CEIB S4-3 exhibits resistance to high concentrations of glyphosate. When exposed to the commercial GBH formulation, the strain tolerated concentrations up to 1600 mg/L, and no growth inhibition was observed across the range of 25–12,000 mg/L in agar plate assays. Minimal inhibition was observed in liquid culture (trypticase soy broth), with results similar to those obtained from the agar plate assays. At a concentration of 25 mg/L, the bacterial strain showed no inhibition, and its growth profile remained comparable to that observed in the absence of glyphosate. Furthermore, *C. zhejiangensis* CEIB S4-3 was capable of degrading up to 61.1 ± 2.2% of glyphosate. Genomic analysis of the strain revealed the presence of key genes associated with two well-established glyphosate degradation pathways, the sarcosine and AMPA pathways, suggesting that the strain utilizes both metabolic routes for glyphosate degradation. Based on these findings, *C. zhejiangensis* CEIB S4-3 presents strong potential as a candidate strain for developing strategies aimed at the biodegradation of glyphosate and AMPA, offering promising applications for mitigating glyphosate contamination in agricultural environments.

## Data Availability

The original contributions presented in this study are included in the article. Further inquiries can be directed to the corresponding author.
